# Safety of catheter ablation of atrial fibrillation without pre‐ or peri‐procedural imaging for the detection of left atrial thrombus in the era of uninterrupted anticoagulation

**DOI:** 10.1002/joa3.12466

**Published:** 2020-12-11

**Authors:** Michael Efremidis, George Bazoukis, Konstantinos Vlachos, Efstathia Prappa, Athanasia Megarisiotou, Stylianos Dragasis, F. Daniel Ramirez, Felix Bourier, Panagiotis Mililis, Athanasios Saplaouras, Gary Tse, Tong Liu, Theodore Efremidis, Panagiotis Kitsoulis, Costas Thomopoulos, Antonios Sideris, Konstantinos P. Letsas

**Affiliations:** ^1^ Second Department of Cardiology Laboratory of Cardiac Electrophysiology "Evangelismos" General Hospital of Athens Athens Greece; ^2^ Hôpital Cardiologique du Haut Lévèque CHU de Bordeaux and IHU‐LIRYC Pessac France; ^3^ Department of Electrophysiology German Heart Center Technical University Munich Germany; ^4^ Xiamen Cardiovascular Hospital Xiamen University Xiamen People's Republic of China; ^5^ Tianjin Key Laboratory of Ionic‐Molecular Function of Cardiovascular Disease Department of Cardiology Tianjin Institute of Cardiology Second Hospital of Tianjin Medical University Tianjin People's Republic of China; ^6^ Laboratory of Anatomy‐Histology‐Embryology School of Medicine University of Ioannina Ioannina Greece; ^7^ Department of Cardiology Helena Venizelou Hospital Athens Greece

**Keywords:** atrial fibrillation, catheter ablation, left atrial thrombus, stroke, transesophageal echocardiography

## Abstract

**Background:**

The need for pre‐ or peri‐procedural imaging to rule out the presence of left atrial thrombus in patients undergoing catheter ablation of atrial fibrillation (AF) is unclear in the era of uninterrupted direct oral anticoagulant (DOAC) regimen. We sought to examine the safety of catheter ablation in appropriately selected patients with paroxysmal AF without performing screening for left atrial thrombus.

**Patients and Methods:**

Consecutive patients planned for radiofrequency AF catheter ablation between January 2016 and June 2020 were enrolled, and prospectively studied. All subjects were receiving uninterrupted anticoagulation with DOACs for at least 4 weeks before the procedure. All subjects were in sinus rhythm the day of the procedure. The primary outcome of the study was ischemic stroke or transient ischemic attack (TIA) during at 30 days.

**Results:**

A total of 451 patients (age 59.7 ± 10.2 years, 289 males) with paroxysmal AF were included in the study. The mean CHA_2_DS_2_‐VASc score was 1.4 ± 1.2. The mean left ventricular ejection fraction and left atrial diameter were 60 ± 5% and 39.3 ± 4 mm, respectively. Regarding the anticoagulation regimen, apixaban was used in 197 (43.6%) patients, rivaroxaban in 148 (32.8%) patients, and dabigatran in 106 (23.5%) patients. None of the patients developed clinical ischemic stroke or TIA during the 30‐day post‐discharged period.

**Conclusions:**

Catheter ablation can be safely performed in low‐risk patients with paroxysmal AF without imaging for the detection of left atrial thrombus in the era of uninterrupted DOAC anticoagulation.

## Graphical abstract

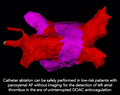

## 1 INTRODUCTION

Transesophageal echocardiography (TOE) is considered the gold standard for the assessment of preexisting left atrial (LA) thrombus in the setting of atrial fibrillation (AF) catheter ablation (CA).^1^ Intracardiac echocardiography or cardiac computed tomography are also used, but less commonly, for this reason.^2^, ^3^, ^4^ Based on the most recent expert consensus statement on AF CA, TOE is reasonable in patients who present in sinus rhythm and have not been anticoagulated prior to CA and in patients who are in AF and have been receiving anticoagulation for at least 3 weeks before the procedure (Class IIa indication).^1^ However, in this consensus statement, only 51% of the Task Force members perform TOE in all patients undergoing AF CA, irrespective of the presenting rhythm and the anticoagulation treatment.^1^

Initial AF CA studies with an interrupted periprocedural anticoagulation strategy with vitamin K antagonists (VKAs) question the use of preprocedural TOE in patients undergoing AF CA.^5^, ^6^ Recent randomized trials have demonstrated the safety of uninterrupted direct oral anticoagulants (DOACs) as compared to uninterrupted VKAs in patients undergoing AF CA.^7^, ^8^, ^9^ In a recent meta‐analysis, uninterrupted periprocedural DOACs have been associated with a lower incidence of cerebrovascular events as compared with uninterrupted VKAs.^10^ Whether adoption of an uninterrupted anticoagulation strategy with DOACs obviates the need of TOE or other imaging to screen for the presence of LA thrombus in adequately anticoagulated patients undergoing AF CA remains a knowledge gap. Previous reports addressing this issue have incorporated intracardiac echocardiography or cardiac computed tomography.^2^, ^3^, ^4^ In this prospective study, we sought to examine the safety of paroxysmal AF CA in adequately anticoagulated patients with DOACs without performing pre‐ or peri‐procedural screening for left atrial thrombus.

## 2 METHODS

### 2.1 Patient population

Consecutive patients planned for radiofrequency AF CA between January 2016 and June 2020 were enrolled, and prospectively studied. Patient demographics, medical history, medications, and echocardiographic data (ie, left ventricular ejection fraction [LVEF], LA diameter, and the presence of valvular heart disease) were collected in all cases. The thromboembolic risk was estimated with the CHA_2_DS_2_‐VASc score. Written informed consent was obtained from all participants, according to institutional guidelines.

### 2.2 Exclusion criteria

Study exclusion criteria included AF as the presenting rhythm at the day of the procedure, persistent or long‐standing persistent AF, previous LA ablation procedures, inadequate anticoagulation for ≥4 weeks prior to the ablation procedure, VKAs use, history of LA thrombus, previous stroke or transient ischemic attack (TIA), history of end‐stage renal disease, structural heart disease (LVEF <45%, hypertrophic cardiomyopathy, severe valvular heart disease, and congenital heart disease), and LA enlargement >45 mm. Patients that did not complete the predefined follow‐up were also excluded from the analysis.

### 2.3 Periprocedural anticoagulation and catheter ablation procedure

All subjects were receiving uninterrupted anticoagulation with DOACs (apixaban 5 mg twice daily, rivaroxaban 20 mg once daily, or dabigatran 150 mg twice daily) for at least 4 weeks before the procedure. Reduced doses of DOACs were prescribed according to drug‐dosage instructions. The last dose of rivaroxaban was taken on the evening prior to the procedure, whereas the last doses of apixaban and dabigatran were taken on the morning of the procedure. During the procedure, we maintained an activated clotting time between 300 and 400 seconds using intravenous administration of unfractionated heparin. Sheaths were irrigated with heparinized normal saline throughout the procedure. DOACs were readministered 3‐4 hours after the procedure and continued for at least 3 months or longer, based on the CHA_2_DS_2_‐VASc score.

The ablation procedure has been described in details elsewhere.^11^ In brief, following a single transseptal puncture, the three‐dimensional geometry of the LA was reconstructed using the CARTO 3 navigation system (Biosense Webster, Inc). All subjects underwent the same ablation protocol including wide circumferential pulmonary vein antral isolation (PVAI) around ipsilateral pulmonary veins (PVs) using a Thermocool SmartTouch irrigated‐tip contact force‐sensing ablation catheter (Biosense Webster, Inc). Radiofrequency energy was applied for 15‐20 seconds at each point using a maximum power of 40 W for the anterior wall and 30‐40 W for the posterior wall, maximum temperature of 42°C, and flow rate of 17 mL/min. Operators attempted to maintain contact force values between 8 and 20 g throughout the procedure. Complete PVAI (entrance and exit block) was confirmed in all cases after a waiting period of 30 minutes. No lines or electrograms‐based ablation were performed in this series of patients.

### 2.4 Post‐ablation care and follow‐up

Patients were hospitalized for 24 hours after CA. Transthoracic echocardiography was performed after the procedure to rule out the presence of pericardial effusion. A detailed neurological examination was performed in all subjects before discharge in order to exclude ischemic stroke or TIA. Additionally, a post‐discharged 30‐day outpatient clinic examination was carried out.

### 2.5 Outcomes

The primary outcome was incident ischemic stroke (defined as a neurologic deficit lasting >24 hours due to acute focal injury of the central nervous system [brain, retina, or spinal cord]) or TIA (defined as a transient episode of neurologic dysfunction with spontaneous clinical resolve within 24 hours) during the 30‐day post‐discharged period.

### 2.6 Statistical analysis

Continuous variables were presented as mean values ± standard deviation, while categorical ones were presented as absolute and relative frequencies (percentages). Analyses were done with SPSS (version 17.0, SPSS Inc).

## 3 RESULTS

The initial study population included 1280 patients referred for AF CA. After applying the exclusion criteria, a total of 451 patients with paroxysmal AF (mean age 59.7 ± 10.2 years old, 289 males) were enrolled in a prospective manner. The clinical, echocardiographic, procedural, and medication data of the study cohort are depicted in Table 1. The mean CHA_2_DS_2_‐VASc score was 1.4 ± 1.2. Specifically, 116 (25.7%) patients displayed CHA_2_DS_2_‐VASc score 0, 138 (30.6%) patients 1, 114 (25.3%) 2, 54 (12%) 3, 24 (5.3%) 4, 4 (0.9%) 5, and 1 (0.2%) 6. The mean LVEF and LA diameter were 60 ± 5% and 39.3 ± 4 mm, respectively. All subjects were in sinus rhythm the day of the procedure. Regarding the anticoagulation regimen, apixaban was used in 197 (43.6%) patients, rivaroxaban in 148 (32.8%) patients, and dabigatran in 106 (23.5%) patients. There were no procedural‐related deaths, while four (0.9%) patients experienced a complication. Specifically, one (0.2%) patient exhibited cardiac tamponade that was managed with pericardiocentesis, two (0.4%) patients developed mild pericardial effusion that was treated conservatively, and one (0.2%) patient had a femoral arteriovenous fistula at the puncture site. None of the patients developed clinical ischemic stroke or TIA during the 30‐day post‐discharged period.

**TABLE 1:** Baseline characteristics and outcomes of the included patients

Variables	n = 451 patients
Clinical data	
Age (years)	59.7 ± 10.2
Sex (males)	289 (64.1%)
CHA_2_DS_2_‐VaSC score	1.4 ± 1.2
Hypertension (%)	220 (48.8%)
Diabetes mellitus (%)	36 (8%)
Dyslipidemia (%)	145 (32.2%)
AF duration (years)	4.3 ± 3.5
Echocardiographic and procedural data	
LVEF (%)	60 ± 5
LAD (mm)	39.3 ± 4
Procedure duration (min)	119.9 ± 58.3
Anticoagulation regimen	
Rivaroxaban (%)	148 (32.8%)
Apixaban (%)	197 (43.6%)
Dabigatran (%)	106 (23.5%)
Other medications	
Class IC antiarrhythmic drugs (%)	207 (45.9%)
Class III antiarrhythmic drugs (%)	73 (16.2%)
β‐blockers (%)	176 (39%)
ACEi/ARBs (%)	178 (39.5%)
Statins (%)	164 (36.4%)
Procedural‐related complications	
Small pericardial effusion (%)	2 (0.4%)
Femoral arteriovenous fistula (%)	1 (0.2%)
Cardiac tamponade (%)	1 (0.2%)
Stroke/TIA (%)	0 (0%)

Abbreviations: ACEi, angiotensin‐converting enzyme inhibitor; ARBs, angiotensin II receptor blockers; LAD, left atrial diameter; LVEF, left ventricular ejection fraction; TIA, transient ischemic attack.

## 4 DISCUSSION

This single‐center study demonstrated the proof‐of‐concept that AF CA can be safely performed without preprocedural imaging in patients with paroxysmal AF in the setting of uninterrupted DOAC anticoagulation. None of the patients meeting these criteria developed an ischemic stroke or TIA during 30‐days of follow‐up.

The utility of preprocedural imaging with TOE in patients undergoing AF CA has been initially investigated in studies incorporating an interrupted anticoagulation strategy. In a study of interrupted anticoagulation strategy (warfarin with periprocedural bridging therapy with enoxaparin), the incidence of LA thrombus detected by TOE was 1.47%.^5^ Persistent AF, female sex, structural heart disease, and LA enlargement were predictors of LA thrombus. Of note, all patients with LA thrombus had persistent or long‐standing AF and dilated LA. In a similar study, the prevalence of LA thrombus was 1.9%.^6^ Hypertension, older age and structural heart disease have been associated with thrombus formation. No thrombus were detected in patients without clinical risk factors.^6^ In addition, the CHA_2_DS_2_VASc score has been shown to identify patients that do not require TOE prior to AF CA in a study adopting an interrupted anticoagulation strategy. None of the patients with a score of 0 or 1 displayed LA thrombus.^12^ Based on these initial reports incorporating an interrupted anticoagulation approach with VKAs, patients with LA thrombus commonly display certain high‐risk features.

During the last decade the periprocedural anticoagulation of AF CA has been considerably changed. The interrupted approach has been abandoned and the DOACs is the preferred anticoagulation regimen. The advent of the uninterrupted anticoagulation therapy as a recommended protocol, significantly reduced the periprocedural stroke and bleeding complications compared with bridging therapy.^13^ Recent randomized studies have demonstrated the excellent safety profile of uninterrupted DOACs as compared to uninterrupted warfarin in patients undergoing AF CA.^7^, ^8^, ^9^ Of note, in RE‐CIRCUIT (dabigatran) and VENTURE‐AF (rivaroxaban) trials, thromboembolic events occurred only in the warfarin group.^7^, ^8^ In a large cohort of 6186 patients with an average CHA_2_DS_2_‐VASc score of 2.86 ± 1.58 who underwent AF CA on uninterrupted DOAC regimen for at least 4 weeks before the procedure, intracardiac echocardiography ruled out the LA thrombus in all patients and revealed “smoke” in 1672 (27.03%) patients.^2^ TIA was observed in one patient with persistent AF, in the setting of a missed dose of rivaroxaban prior to ablation. This study mainly underscores the excellent safety profile of uninterrupted DOAC strategy at least 4‐weeks before the procedure in high stroke risk patients referred for AF CA. However, even in this study, intracardiac echocardiography was used as an alternative to TOE. Di Biase et al enrolled mainly persistent AF patients who underwent AF CA while on uninterrupted apixaban and rivaroxaban regimen.^3^ Intracardiac echocardiography was performed in all patients to exclude LA thrombus, but in only 71% of them the LA appendage was visualized. In the rest of the patients (29%), AF ablation was performed without imaging of the LA appendage. In this study, only one (0.1%) patient with long‐standing AF suffered a TIA. In a study assessing the trends in TOE use since transition to a strategy of uninterrupted warfarin or briefly interrupted DOAC therapy, the use preprocedural TOE dropped significantly throughout a 5‐year period, from 86% to 42%, without an increase in thromboembolic events.^14^ Dense spontaneous echo contrast and LA thrombus were associated with persistent AF, higher CHA_2_DS_2_VASC score, increased LA size, reduced LA appendage flow velocity, and decreased LVEF. Preprocedural TOE may be practically avoided in patients without high‐risk features.^14^

Up to now, there is only one report addressing the safety of AF CA without pre‐ or peri‐procedural imaging.^15^ In this study, 1231 patients underwent AF CA on uninterrupted DOAC and the presenting rhythm was AF or atrial flutter. Thromboembolic complications occurred in 5 (0.4%) patients.^15^ Our study differs with respect to patient selection offered an AF CA without pre‐ or peri‐procedural imaging. We demonstrated the excellent safety of AF CA on uninterrupted DOAC regimen without imaging for LA thrombus in a carefully selected population with paroxysmal AF displaying low thromboembolic risk (mean CHA_2_DS_2_‐VASc score of 1.4 ± 1.2) and absence of significant LA enlargement. Based on our findings, patients without risk factors for the presence of LA thrombus such as persistent AF, structural heart disease, and dilated LA,^2^, ^3^, ^5^, ^6^, ^12^, ^13^, ^14^ can safely undergo AF CA without pre‐ or peri‐procedural imaging.

TOE presents a certain risk of complications including injuries of the oral, esophageal, and gastrointestinal tracts, bleeding of esophageal tract, changes in esophageal integrity, dysphagia, or odynophagia, laryngeal palsy, and very rarely death (0.01%‐0.02%).^16^ These complications can be aggravated during CA procedures in the setting of high anticoagulation conditions. Given the near zero rate of thromboembolic events in the era of uninterrupted anticoagulation with DOACs,^7^, ^8^, ^9^ particularly in patients with paroxysmal AF without comorbidities, TOE appears to subject a low‐risk patient to higher risks than benefit. Precautions that minimize fresh thrombus formation during the procedure by adequate anticoagulation is, therefore, more relevant than trying to detect LA appendage thrombus by TOE. In addition, in the era of severe acute respiratory syndrome coronavirus 2 (SARS‐CoV‐2) pandemic (COVID‐19), TOE procedure may expose healthcare workers to an increased risk of SARS‐CoV‐2 infection through respiratory droplets. Finally, a cost‐effectiveness study has demonstrated that routine use of TOE is associated with considerable cost per quality‐adjusted life year.^17^

In conclusion, AF CA can be safely performed without pre‐ or peri‐procedural imaging in the era of uninterrupted DOAC anticoagulation in carefully selected patients with paroxysmal AF.

### 4.1 Limitations

The present report has potential limitations. First, the presence of thromboembolic events was assessed based on clinical examination. Cerebral magnetic resonance imaging was not routinely performed. Second, the number of patients is relatively small. Further studies in larger number of patients are needed to validate our findings. Finally, although patients with AF as the presenting rhythm at the day of the procedure were excluded, LA stunning may be present after recent restoration of sinus rhythm even in the case of paroxysmal AF predisposing these patients to thromboembolic events. Adequate anticoagulation for ≥4 weeks prior to the ablation procedure may overcome this limitation.

## DISCLOSURE

The authors declare no conflicts of interest.

## References

[1] Calkins H , Hindricks G , Cappato R , et al. 2017 HRS/EHRA/ECAS/APHRS/SOLAECE expert consensus statement on catheter and surgical ablation of atrial fibrillation. Europace. 2018;20(1):e1–e160. 10.1093/europace/eux274 PMC5834122 29016840

[2] Patel K , Natale A , Yang R , et al. Is transesophageal echocardiography necessary in patients undergoing ablation of atrial fibrillation on an uninterrupted direct oral anticoagulant regimen? Results from a prospective multicenter registry. Heart Rhythm. 2020:S1547‐5271(20)30672‐X 10.1016/j.hrthm.2020.07.017 32681991

[3] Di Biase L , Briceno DF , Trivedi C , et al. Is transesophageal echocardiogram mandatory in patients undergoing ablation of atrial fibrillation with uninterrupted novel oral anticoagulants? Results from a prospective multicenter registry. Heart Rhythm. 2016;13(6):1197–202. 26994940 10.1016/j.hrthm.2016.03.024

[4] Kottmaier M , Jilek C , Berglar S , et al. Exclusion of left atrial thrombus by dual‐source cardiac computed tomography prior to catheter ablation for atrial fibrillation. Clin Res Cardiol. 2019;108(2):150–6. 30051177 10.1007/s00392-018-1333-0

[5] McCready JW , Nunn L , Lambiase PD , et al. Incidence of left atrial thrombus prior to atrial fibrillation ablation: is pre‐procedural transoesophageal echocardiography mandatory? Europace. 2010;12(7):927–32. 20304842 10.1093/europace/euq074

[6] Calvo N , Mont L , Vidal B , et al. Usefulness of transoesophageal echocardiography before circumferential pulmonary vein ablation in patients with atrial fibrillation: is it really mandatory? Europace. 2011;13(4):486–91. 21186230 10.1093/europace/euq456

[7] Cappato R , Marchlinski FE , Hohnloser SH , et al.; VENTURE‐AF Investigators . Uninterrupted rivaroxaban vs. uninterrupted vitamin K antagonists for catheter ablation in non‐valvular atrial fibrillation. Eur Heart J. 2015;36(28):1805–11. 25975659 10.1093/eurheartj/ehv177 PMC4508487

[8] Calkins H , Willems S , Gerstenfeld EP , et al.; RE‐CIRCUIT Investigators . Uninterrupted dabigatran versus warfarin for ablation in atrial fibrillation. N Engl J Med. 2017;376(17):1627–36. 28317415 10.1056/NEJMoa1701005

[9] Kloosterman M , Chua W , Fabritz L , et al.; AXAFA‐AFNET 5 Investigators . Sex differences in catheter ablation of atrial fibrillation: results from AXAFA‐AFNET 5. Europace. 2020;22(7):1026–35. 32142113 10.1093/europace/euaa015 PMC7336181

[10] Cardoso R , Knijnik L , Bhonsale A , et al. An updated meta‐analysis of novel oral anticoagulants versus vitamin K antagonists for uninterrupted anticoagulation in atrial fibrillation catheter ablation. Heart Rhythm. 2018;15(1):107–15. 28917562 10.1016/j.hrthm.2017.09.011

[11] Efremidis M , Vlachos K , Letsas KP , Bazoukis G , Martin R , Frontera A , et al. Targeted ablation of specific electrogram patterns in low‐voltage areas after pulmonary vein antral isolation in persistent atrial fibrillation: termination to an organized rhythm reduces atrial fibrillation recurrence. J Cardiovasc Electrophysiol. 2019;30(1):47–57. 30288830 10.1111/jce.13763

[12] Atkinson C , Hinton J , Gaisie EB , et al. Use of the CHA _2_ DS _2_ VASc score to reduce utilisation of transoesophageal echocardiography prior to ablation for atrial fibrillation. Echo Res Pract. 2017;4(4):45–52. 28864464 10.1530/ERP-17-0042 PMC5633057

[13] Di Biase L , Burkhardt JD , Santangeli P , Mohanty P , Sanchez JE , Horton R , et al. Periprocedural stroke and bleeding complications in patients undergoing catheter ablation of atrial fibrillation with different anticoagulation management: results from the Role of Coumadin in Preventing Thromboembolism in Atrial Fibrillation (AF) Patients Undergoing Catheter Ablation (COMPARE) randomized trial. Circulation. 2014;129(25):2638–44. 24744272 10.1161/CIRCULATIONAHA.113.006426

[14] Balouch M , Gucuk Ipek E , Chrispin J , Bajwa RJ , Zghaib T , Berger RD , et al. Trends in transesophageal echocardiography use, findings, and clinical outcomes in the era of minimally interrupted anticoagulation for atrial fibrillation ablation. JACC Clin Electrophysiol. 2017;3(4):329–36. 29759444 10.1016/j.jacep.2016.09.011

[15] Diab M , Wazni OM , Tarakji K , et al. Safety of radiofrequency ablation without pre‐procedural trans‐esophageal echocardiography in patients with atrial fibrillation on novel oral anticoagulants. Circulation. 2019;140:A15938.

[16] American Society of Anesthesiologists and Society of Cardiovascular Anesthesiologists Task Force on Transesophageal Echocardiography . Practice guidelines for perioperative transesophageal echocardiography. An updated report by the American Society of Anesthesiologists and the Society of Cardiovascular Anesthesiologists Task Force on Transesophageal Echocardiography. Anesthesiology. 2010;112(5):1084–96. 20418689 10.1097/ALN.0b013e3181c51e90

[17] Gula LJ , Massel D , Redfearn DP , Krahn AD , Yee R , Klein GJ , et al. Impact of routine transoesophageal echocardiography on safety, outcomes, and cost of pulmonary vein ablation: inferences drawn from a decision analysis model. Europace. 2010;12(11):1550–7. 20716548 10.1093/europace/euq306

